# Nucleoside phosphorylation by the mineral schreibersite

**DOI:** 10.1038/srep17198

**Published:** 2015-11-26

**Authors:** Maheen Gull, Mike A. Mojica, Facundo M. Fernández, David A. Gaul, Thomas M. Orlando, Charles L. Liotta, Matthew A. Pasek

**Affiliations:** 1 School of Geoscience, University of South Florida, 4202 E Fowler Ave, Tampa FL 33620; 2 School of Chemistry and Biochemistry, Georgia Institute of Technology, 901 Atlantic Drive, Atlanta GA 30332.

## Abstract

Phosphorylation of the nucleosides adenosine and uridine by the simple mixing and mild heating of aqueous solutions of the organic compounds with synthetic analogs of the meteoritic mineral schreibersite, (Fe,Ni)_3_P under slightly basic conditions (pH ~9) is reported. These results suggest a potential role for meteoritic phosphorus in the origin and development of early life.

An RNA-based biochemistry likely preceded modern DNA-protein-based biochemistry^1^,^2^. The construction of RNA presently requires activation of nucleotides by biosynthesis of nucleoside triphosphates, which then assemble to RNA with loss of pyrophosphate. In this respect, phosphorus-containing species may have been important reagents during prebiotic chemical evolution. The low reactivity of phosphates, however, has presented a problem in understanding the origin of phosphorylated biomolecules, such as RNA^1^,^2^. The thermodynamics of phosphorylation reactions in water are endergonic and require a dehydration step^3^, disfavoring phosphorylation. Phosphorylation by phosphate minerals is also inhibited by divalent cations such as Mg^2+^ and Ca^2+^, both of which were likely abundant in early oceans, and which cause precipitation of phosphate from water^4^. Phosphorylation of organic compounds by phosphate minerals have thus been performed in anhydrous solvents^5^, by heating well above the boiling point of water^6^, by addition of a reactive reagent such as cyanate^7^, or by adsorption of the reagents on minerals^8^. Alternatively, reactive condensed phosphates such as trimetaphosphate are capable of phosphorylating simple organics^9^, though there are few natural sources of such species, and many of these reactions still require elevated temperatures.

Although phosphorylation can take place using a variety of phosphate minerals in non-aqueous solvents^5^, prebiotic phosphorylation in water is more likely given the dominance of water as a solvent across the Solar System. Highly soluble phosphate minerals increase orthophosphate availability and thereby increase reaction rates. However, not all phosphate minerals known today were present on the early earth, as minerals have evolved with life and with the development of plate tectonics^10^,^11^. The most soluble phosphate minerals are mostly biological in origin and hence originated well after prebiotic chemistry^10^. Given the presumed high flux of meteoritic material to the early earth^12^,^13^, the meteoritic phosphide mineral schreibersite (Fe,Ni)_3_P would have also been present on the earth’s surface^14^. Although the exact contribution of meteoritic phosphorus to the total phosphorus budget is uncertain, Archean carbonates show a meteoritic signature for phosphorus in the early oceans^15^. Up to 10% of the earth’s crustal phosphorus may have originated from schreibersite, hence this mineral was readily available to engage in early chemical reactions^14^,^16^.

Herein we report the phosphorylation of the nucleosides uridine and adenosine using synthetic schreibersite analogs (Fe_3_P and Fe_2_NiP) as the phosphorus source. Phosphorylation of glycerol by Fe_3_P to give glycerol phosphate (a component of cell membranes) has been reported previously^15^; however, the free energy required to phosphorylate glycerol is about half that of other potential prebiotic molecules, such as adenosine^14^. A thorough exploration of the extent of phosphorylation of nucleosides by schreibersite and its analogs is necessary to evaluate its potential prebiotic importance. To this end, aqueous solutions of nucleosides were sealed in vials with Fe_3_P or Fe_2_NiP, and stirred and heated to 80 °C for 2 days to 2 weeks (see SI for details). Urea, bases including K_2_CO_3_ and NH_4_OH, and/or MgSO_4_ were added to some reaction mixtures to probe the effects of their presence on phosphorylation yield^17^,^18^.

The resulting solutions were analyzed by UPLC-MS, MS/MS, and ^31^P NMR to determine the extent of production of phosphorylated compounds. Quantification was performed by external calibration with synthetic standards of phosphorylation reaction products. The synthetic schreibersite (Fe_3_P or Fe_2_NiP) used in this study was determined to be structurally identical to its meteoritic counterpart by X-ray diffractometry, X-ray photoelectron spectroscopy, and Raman spectroscopy^19^, and compositionally identical to some forms of naturally occurring schreibersite^20^,^21^.

Nucleosides were successfully phosphorylated by Fe_3_P, and Fe_2_NiP (Figs 1 and 2 and Table 1). Identity of reaction products was confirmed by accurate mass measurements and matching of chromatographic elution time and MS/MS fragmentation spectra against standards. Nucleotides were produced at concentrations ranging between 1 to 6% of total dissolved P in those solutions where they were detected (Table 1); and in the case of adenosine phosphorylation by Fe_3_P in the presence of urea and MgSO_4_, this result was further confirmed by NMR (Fig. 3). Although the yields of phosphorylated products were low, it appeared that the pH of the aqueous phase was a dominant variable in the phosphorylation outcomes. All experiments indicated that a basic pH was required for the production of phosphorylated products. Phosphorylation yields appeared to be somewhat proportional to the amount of phosphorus dissolved in solution and potentially independent of the presence of nickel in the starting schreibersite simulant.

Figure 1 provides extracted ion chromatograms at *m/z* 346.05 (with a 0.06 *m/z* window). Since 2′-, 3′-, and 5′-adenosine monophosphate are isomers, the elution times and mass fragmentation patterns (Fig. 2) were compared to standards for identification purposes. Additionally, ^31^P NMR spectra revealed a triplet corresponding to a 3-bond interaction between two H atoms and the P atom, and two doublets corresponding interactions between a single H atom interacting with a P atom 3 bonds away (Fig. 3). Standard calibration curves were generated to quantitate phosphorylated products by UPLC-MS. These product masses were contrasted to total dissolved P measured by ICP-OES (Table 1).

It was observed that no detectable phosphorylation of adenosine with Fe_3_P took place in deionized water at a pH of 6.5 after 14 days. In addition, only small quantities of inorganic phosphorus species were detected in solution (Table 1, Entry 1). In the presence of urea over the same period of time, however, adenosine phosphorylation was observed and the amount of inorganic phosphorus species in solution increased by a factor of 10 (Table 1, Entry 2). Two important observations were made: (1) the 5′ phosphorylated product dominated the product suite and (2) the pH of the aqueous solution increased from 6.5 to 9.5. In the presence of both urea and magnesium sulfate both the nucleotide products and the inorganic phosphorus species increased (Table 1, Entry 3). The amount of magnesium sulfate used slightly exceeded its solubility in water at 80 °C (0.51g in 7 mL H_2_O at 100 °C)^22^; the reaction mixture was heterogeneous. As in the previous case, the pH again increased from 6.5 to 9.0. It appeared that even in the presence of large amounts of magnesium ion, phosphorylation of adenosine was still observed and was even enhanced compared to experiments containing only urea. This was surprising since divalent cationic species typically precipitate phosphate, and the concentration of Mg^2+^ added exceeded the solubility of both magnesium phosphate and magnesium phosphite. This result could imply that the phosphorylating agent is possibly a phosphorus species in an oxidation state other than +5 and thus would have sufficient aqueous solubility in the presence of a divalent cation. It was also conjectured that the pH drift from “neutral” to basic was due to the hydrolysis of urea and the formation of ammonium carbonate. Indeed, in the presence of potassium carbonate or ammonium hydroxide (pH 12.5 and pH 11.5, respectively) in place of urea, phosphorylation of adenosine was also observed (Table 1, Entries 7 and 8). Again, the 5′ product dominated. It was concluded that urea itself does not take a direct role in the phosphorylation process. It is merely the precursor in attaining a basic pH. A similar set of observations were made in the phosphorylation reactions of uridine (Table 1, Entries 4, 5, and 6). In these cases, however, the selectivity of the 5′ product does not appear as efficient as in the adenosine case. It should be noted, however, that the 2′ and 3′ phosphorylated isomers could not be separated by UPLC.

The mechanism of phosphorylation is still unknown and is being actively investigated. It is possible that the process occurs in solution or on the surface of the schreibersite. As was mentioned previously the yield of organophosphates from these experiments is not high, and the concentration of AMP ranges from being undetectable to about 600 μM. The yield seems to be controlled by the release of P from phosphide corrosion, which is slow^23^, and since the amount of organic substrate and initial Fe_3_P added essentially does not change, the measured yield is dependent on reaction conditions influencing P release. The amount of phosphide that corroded over the experimental timescales is between 0.05% (without additives) and up to 9.5% (with K_2_CO_3_), in agreement with measured corrosion rates of 0.1% per week without added solutes^23^,^24^. If yields are calculated with respect to added nucleoside, they are lower as the nucleoside is likely not the limiting reagent in this reaction.

To determine the relevance of our model environment (schreibersite corroding in an organic-rich aqueous solution) to prebiotic chemistry, the steady state concentration of nucleotides is equal to the production rate divided by the hydrolysis rate constant. In the absence of biological enzymes hydrolyzing these organophosphates, the rate of hydrolysis is typically slow (~10^−7^ s^−1^, SI), suggesting an accumulation of up to a few mM at steady state for up to 10 years, under these conditions. The production rate is estimated from the measured concentration of AMP from the experiments over the 2 to 14 day period (Table 1). It is unknown whether these concentrations of organophosphates would be sufficient to lead to biopolymer development over these timescales.

Prior work on nucleoside phosphorylation has shown that inorganic phosphate can serve as both a catalyst and reactant in nucleotide synthesis^25^. The oxidation of schreibersite by water is the ultimate source of a variety of soluble phosphorus compounds, and generates iron and nickel oxides and hydroxides^24^,^25^, as well as H_2_ gas^26^. In lieu of direct corrosion to insoluble phosphate minerals, schreibersite instead oxidizes to form a mixed-valence suite of phosphorus oxyanions, including phosphite, hypophosphate, pyrophosphate and orthophosphate, and these anions are free to react both in solution and possibly by surface-mediated chemistry. The oxidation of schreibersite to magnetite (Fe_3_O_4_) and phosphorus oxyanions is a strongly exergonic process releasing 100 kJ·mol^−1^ at 80 °C and it is possible that some portion of this energy is directed to phosphorylation.

Although O_2_ was not excluded from the experiments, prior work^24^,^26^,^27^ has shown little variation in P speciation when performed under an oxygenated atmosphere. The total quantity of O_2_ in the headspace of these experiments or dissolved within the solutions was insufficient to promote oxidation of the schreibersite to release the concentrations of P observed.

A source of reactive phosphorus may have been an important part of the prebiotic earth, and possibly of Mars^28^. Life today builds RNA from activated nucleotides, and phosphate and its organic derivatives are an important part in metabolic processes^29^, with about half of all metabolic processes involving a phosphorylated biomolecule in some form. In this respect, phosphorylated biomolecules likely played an important part in the prebiotic chemical milieu from which life emerged. Since schreibersite analogs are capable of phosphorylating simple hydroxyl-compounds, it may have been one of the initial phosphorylating reagents leading to the emergence of metabolic molecules such as ATP. Phosphorylation of other molecules by schreibersite is certainly feasible, including the generation of phospholipids^15^. The prebiotic environment directly relevant to the experiments described here would be a warm water environment^30^ with meteorite material, and these reactions are single mixtures and require no additional steps to prepare organophosphates. These reactions show that presumed necessary phosphorylated prebiotic molecules were likely present on the early earth, and that the earth was predisposed to phosphorylated biomolecules.

## Methods

For each reaction, the typical quantities for the reagents were as follows; Fe_3_P (0.7 g), and for the other reagents (if used); urea (0.50 g), MgSO_4_ (0.75 g), 25% soln. NH_4_OH (0.29 g), K_2_CO_3_ (1.15 g). For sample U4, 1.5 g of Fe_2_NiP was used instead of 0.7 g Fe_3_P. The sample of Fe_2_NiP was subjected to a series of wet/dry cycles (5×), leading to substantial surface corrosion. The reagents were added in a glass vial containing 7 mL of deionized water, to which the nucleoside was added (0.50 g; SI). Reaction vials were tightly sealed and heated at 80 °C from 2 to 14 days. At the completion of reaction the pH of each reaction was measured at the beginning and end of each reaction to changes due to reaction with metal ions, and breakdown of urea.

Other method and instrumentation details are provided in SI^30^.

## Additional Information

**How to cite this article**: Gull, M. *et al*. Nucleoside phosphorylation by the mineral schreibersite. *Sci. Rep*. **5**, 17198; doi: 10.1038/srep17198 (2015).

## Supplementary Material

Supplementary Information

## Figures and Tables

**Figure 1:**
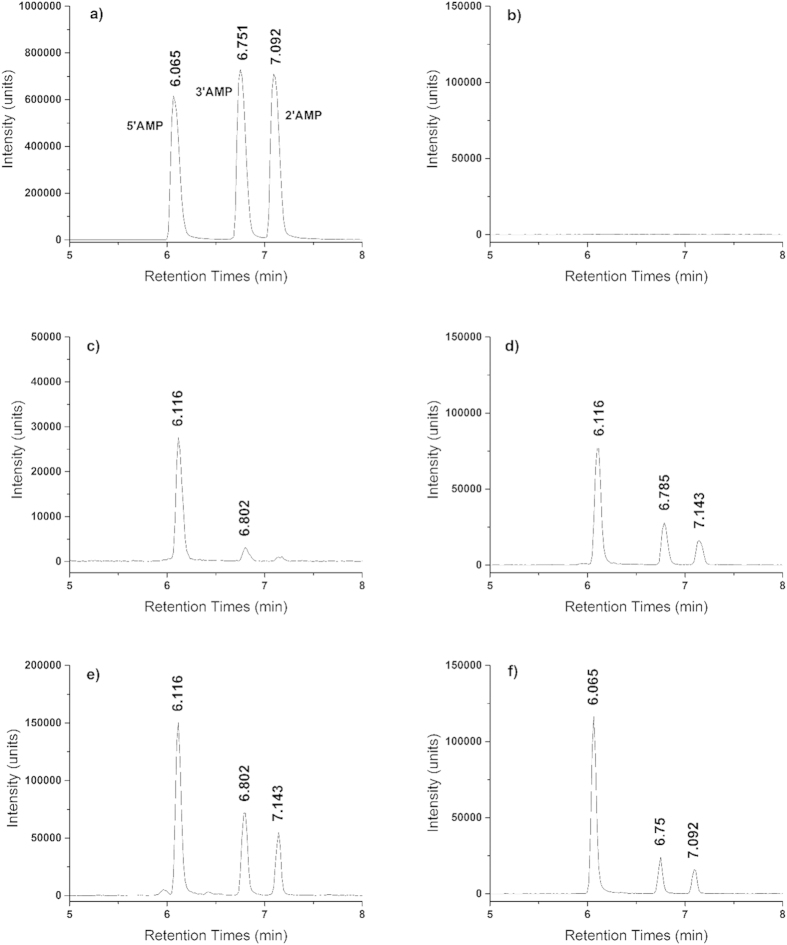
UPLC-MS extracted ion chromatograms at *m/z* = 346.05 ± 0.06, corresponding to adenosine monophosphate [M-H]^−^ ionic species in both a mixture of standards, and in samples Ad1-Ad5. Experimental conditions for Ad1-Ad5 are given in Table 1, and consist of mixtures of adenosine and iron phosphide with other solutes in water, heated to 80 °C. Uridine data may be found in the SI. (**a**) 5′, 3′ and, 2′ AMP standards (50 μM); (**b**) reaction Ad1; (**c**) reaction Ad2; (**d**) reaction Ad3 (**e**) reaction Ad4 (**f**) reaction Ad5.

**Figure 2:**
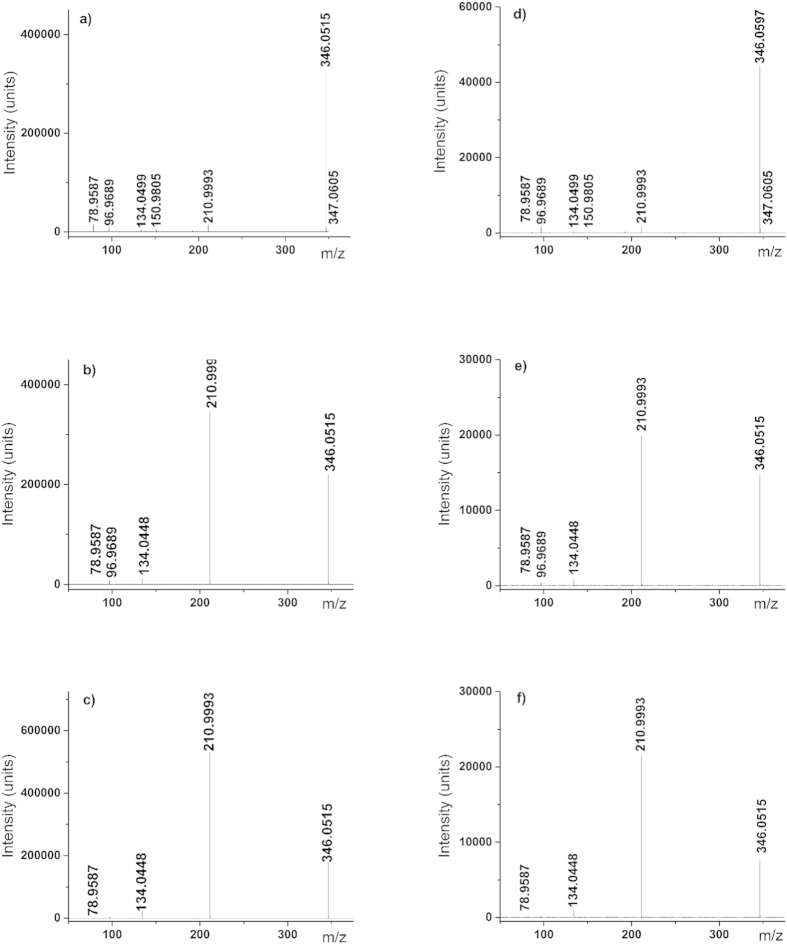
Tandem MS (MS/MS) fragmentation spectra for 5′, 3′, and 2′ adenosine monophosphate standards and corresponding retention-time matched chromatographic peaks in sample Ad4. A collision energy of 15 eV was used. The precursor ion selected in all cases was m/z 346.05. Fragmentation spectra for other reaction mixtures may be found in the SI. (**a**) 50 μM 5′AMP standard (R.T. = 6.06 min); (**b**) 50 μM 3′AMP standard (R.T. = 6.75 min); (**c**) 50 μM 2′ AMP standard (R.T. = 7.09 min); (**d**) reaction Ad4 5′AMP (R.T. = 6.11 min); (**e**) reaction Ad4 3′AMP (R.T. = 6.80 min); (**f**) reaction Ad4 2′AMP (R.T. = 7.14 min).

**Figure 3:**
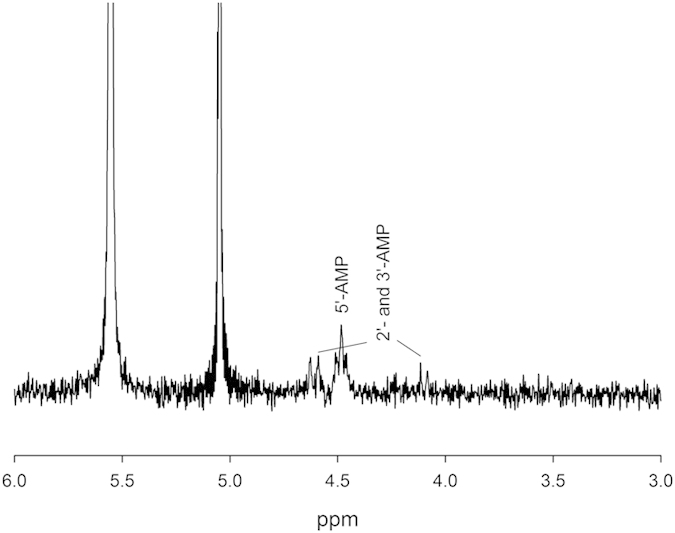
^31^P NMR spectrum of reaction mixture resulting from mixing adenosine with Fe_3_P (equivalent to sample Ad3, but with an additional substrate, FeS; FeS was not found to influence these results). Peaks are identified based on peak position vs. standards, and from J-coupling constants (~5 Hz for 3 bond H-P interactions). The doublets are CH-O-P interactions, and the triplet is a CH_2_-O-P interaction. Species identification was confirmed by spiking with an adenosine monophosphate standard, causing the triplet to increase in signal strength. The peak at 5.1 corresponds to phosphite (HPO_3_^2−^), and the peak at 5.6 is orthophosphate (HPO_4_^2−^). Both compounds are common products during phosphide corrosion^14^, and a majority of the total dissolved P is in these two species.

**Table 1:** Reaction conditions and quantitation of target compounds in synthetic schreibersite reaction mixtures.

Reaction ID (Ad = adenosine, U = uridine)	Initial Reaction Conditions			Phosphorylated nucleotide concentration in μM (Yield based on total P)			Final Reaction Conditions	
	Initial pH	Time (days)	Additional Reagents	*5′*	*3′*	*2′*	Final pH	Total P (mM)
Ad1	6.5	14		ND^a^	ND	ND	6.5	0.24
Ad2	6.5	14	Urea	73 (2.9%)	<5^b^	<5^b^	9.5	2.5
Ad3	6.5	14	MgSO_4_, Urea	210 (4.2%)	41 (0.8%)	38 (0.8%)	9	5
Ad4	12.5	2	K_2_CO_3_	360 (0.8%)	130 (0.3%)	94 (0.2%)	10.5	47
Ad5	11.5	2	NH_4_OH	250 (5.7%)	22 (0.5%)	29 (0.5%)	9.5	4.4
U1	6.5	14		ND	ND		6.5	0.65
U2	6.5	14	Urea	56 (0.6%)	65 (0.7%)		9.5	10
U3	6.5	14	MgSO_4_, Urea	54 (1.5%)	84 (2.4%)		8	3.5
U4	N/A^c^	14	Fe_2_NiP (1.5 g), Urea	200^d^ (0.5%)	~330^d^ (0.8%)		N/A	43

^a^ND: The compound was below the limit of detection (LoD), typically~0.2 μM. ^b^Not quantifiable, below quantitation limit (LoQ). ^c^“N/A” Not Available. ^d^Calculation based on combined area of two partially-resolved peaks. See SI, Figure S3
