# Supplementary material for: Nucleoside phosphorylation by the mineral schreibersite
Source: Sci Rep. 2015 Nov 26;5:17198. doi: 10.1038/srep17198 (PMC4660433; doi:10.1038/srep17198)
Supplement: Supplementary Information [file srep17198-s1.doc]

**Supporting Info: Nucleoside phosphorylation by the mineral schreibersite.**

Maheen Gull^ǂ^, Mike A. Mojica^ǂ^, Facundo M. Fernández, David A. Gaul, Thomas M. Orlando, Charles L. Liotta, & Matthew A. Pasek*

**METHODS (Supplementary Material)**

**Materials**

Adenosine, uridine, urea, Fe_3_P and MgSO_4_ were obtained from Fischer Scientific whereas K_2_CO_3_ and NH_4_OH were obtained from Sigma Aldrich and Na_2_S nonahydrate from EMD chemicals. The iron phosphide was acquired as a -40 mesh powder, corresponding to a surface area of at least 0.001 m^2^/g. Nanopure water (18.2 MΩ cm) from a Barnstead/Thermolyne diamond UV Ultrapure Water System (Dubuque, Iowa) was used to prepare solutions and the mobile phase. Standards of 5’AMP, 3’AMP, 2’AMP (>99%) and 5’UMP (99%) were purchased Sigma. 3’UMP was purchased from Carbosynth Ltd. Analytical reagents included methanol (LC-MS CHROMASOLV®), n-hexylamine (≥99.0%), NH_4_OAc (≥99.7%), and 6N NH_3_ in methanol were purchased from Sigma-Aldrich (St. Louis).

Iron-nickel phosphide was prepared according to the methods of Skála and Drabek (2002), modified with heating under an argon atmosphere (La Cruz 2015). The product was crushed to pass through a 40 sized mesh (surface area >0.001 m^2^/g), and analyzed by a BTX Benchtop XRD (Figure S5). Prior work on this schreibersite analog and on Fe_3_P (Pirim et al. 2014) has demonstrated that this material matches that of natural schreibersite based on Raman spectra, XRD and XPS. A small amount of iron-nickel alloy was found to be a side product of the schreibersite synthesis. However, since schreibersite in meteorites occurs most often in direct contact with iron-nickel metal, this additional product does not change the applicability of these experiments to the prebiotic earth.

**UPLC-MS and MS/MS Analysis**

MS, MS^2^, and ultra-performance liquid chromatography (UPLC) were performed on a Waters Synapt G2 HDMS system in negative-ion, ESI-TOF resolution mode (m/Δm = 20,000 FWHM). A capillary voltage of 2.00 kV was used. For MS^2^, CID fragmentation was performed in the transfer collision cell with collision energies of 15 eV. The UPLC unit was fitted with an Acquity UPLC BEH C18 1.7 µm, 2.1 mm X 50 mm column. An ion pairing procedure was used to separate organophosphates where solvent A was 5 mM aqueous solution of n-hexylamine (pH=11.2) and solvent B was a mixture of 90% methanol and 10% 10 mM NH_4_OAC (pH=8.3). The UPLC flow rate was 0.25 mL min^-1^. A 25 min gradient method was used: 0-2 min, 0 % B; 2-6 min, 0-20 % B; 6-15 min, 20-80 %B, 15-20 min 80-0 %B. Column temperature was maintained at 40°C.

**^31^P NMR Analysis**

Prior to P-NMR analysis, a portion of each of the reaction mixtures was treated with a few drops of 1M Na_2_S until the pH reached 10-11. Thick blackish-brown precipitates were immediately formed. After shaking vigorously, the mixture was filtered and the filtrate (1 mL) was allowed to dry at room temperature (22 ^o^C) for a few hours and subsequently rehydrated with (1 mL) D_2_O (50:50 ratio). If needed, samples were centrifuged.

^31^P NMR spectra were acquired on a Unity INOVA 400 spectrometer (161.84 MHz for ^31^P and 399.882.54 MHz for ^1^H) equipped with a variable temperature controller and a Varian 5mm auto-switchable probe equipped with Z-axis gradient optimized for tuning of ^31^P and ^1^H on the first of a series of samples in D_2_O. The ^31^P chemical shifts are reported using an external reference standard (neat solution of phosphoric acid at room temperature (~25^o^C, δppm=0.0). A ^31^P 45^o^ flip angle pulse was used for both proton decoupled and non-decoupled spectra (90^o^ ^31^P pulse of 9.8 μsec at 54 dB attenuation, were max power output ~300 Watts). For proton-decoupled spectra a composite pulse Waltz decoupling sequence was applied with field strength of 2525 Hz during the acquisition time of 1 s and the relaxation time of 1 s. Quantitative ^31^P NMR was used to determine the hydrolysis rate under these experimental conditions, and the relaxation time was 30 s. The signal was averaged from 512 transients. All spectra were processed by ACD Labs 12.0 package and or vnmnrj 2.2D or 3.2A. Compounds were identified based on J-coupling constants, peak positions, and comparison to standards.

**ICP-OES Analysis**

Samples were analyzed for total phosphorus in solution with a Perkin Elmer Optima 2000DV inductively-coupled plasma-optical emission spectrometer (ICP-OES). The instrument utilizes an Echelle monochromator and employs a CCD detection system to permit rapid resolution of optical interferences. The ICP-OES instrument operated at an RF power of 1300 Watts, with a nebulizer flow of 0.8 mL minute^-1^, auxiliary gas flow of 0.2 mL minute^-1^, and plasma gas flow of 15 mL min^-1^. Samples were diluted by a factor of 50, and compared with OES results from standard solutions. Calibration was done using standards consisting of 0.04, 0.2, 1, 4, and 10 ppm total phosphorus (as phosphate) synthetic standards. Correlation coefficients for the standard curves were 0.9999 or better. An internal quality control standard of 1.2 ppm had an error between separate runs of 6.58%.

**Hydrolysis Rate Determination**

The hydrolysis of AMP was tested in a solution without the potential for further phosphorylation, using a reaction setup that replaced Fe_3_P with Fe metal. 0.5 g of Na_2_AMP salt were added to 7 mL water with 0.5 g of urea, and 0.75 g of Fe metal powder. The solution was heated to 80°C for 7 days, then analyzed by quantitative ^31^P NMR (Figure S1). Hydrolysis products were phosphate (9%) and pyrophosphate (6%). The reaction rate was assumed to be first order with respect to AMP, giving a hydrolysis rate constant *k* of 2 ×10^-7^ s^-1^.

**Figure S1.** Coupled quantitative NMR spectrum of AMP heated to 80°C for 7 days with urea and iron metal. From right to left the peaks correspond to orthophosphate, AMP, and pyrophosphate.


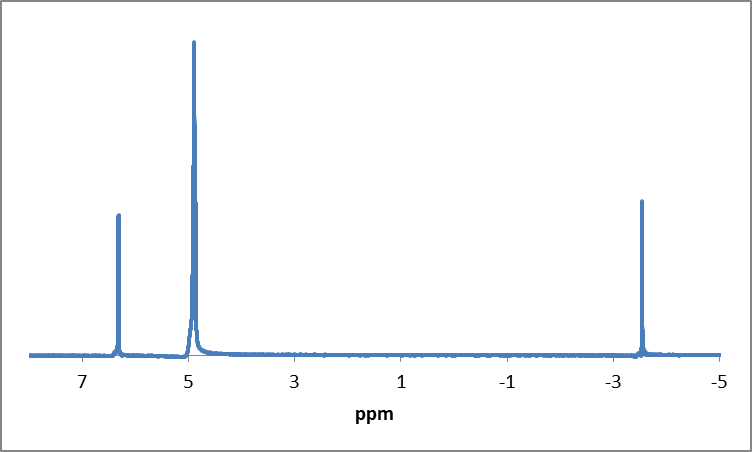


**Figure S2.** MS/MS fragmentation spectra for adenosine + Fe_3_P experiments described in Table 1 (Ad1, Ad2, Ad3, Ad5 from top to bottom). Right to left are 5’, 3’, and 2’ AMP, respectively.

**Figure S3.** UPLC-MS extracted ion chromatograms at *m/z*= 323.03 ± 0.06, corresponding to uridine monophosphate [M-H]^-^. a) 5’ and 3’ UMP standards (50 uM); b) reaction Ur1 c) Reaction Ur2 d) Reaction Ur3 e) Reaction Ur4.

**Figure S4.** MS/MS fragmentation spectra for uridine + Fe_3_P experiments. 5’-UMP is the right column 3’- and 2’- UMP are on the left. From top to bottom: standard mixture, reaction Ur2, Ur3, Ur4.

**Figure S5.** XRD of synthesized schreibersite (pink) with comparison of characteristic d values for schreibersite (gray bars). Other peaks correspond to matches to kamacite, an iron-nickel mineral.**
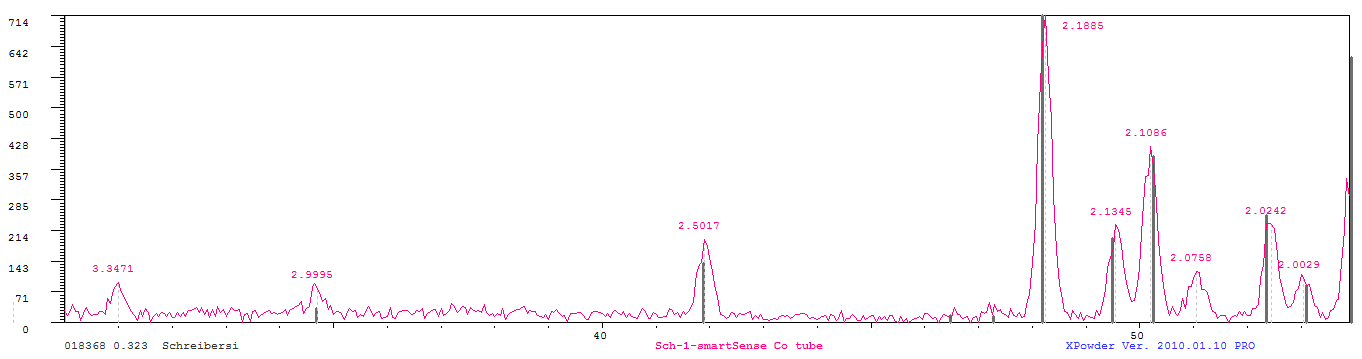
**

References

La Cruz, Nikita. Schreibersite: Synthesis, Characterization and Corrosion and Possible Implications for Origin of Life (2015). MS Thesis, University of South Florida.

Skála, Roman, and Milan Drábek. Powder data for synthetic analogue of a mineral nickelphosphide. *Powder Diffraction* 17.04 (2002): 322-325.
